# “Clinical significance of multifocal and multicentric breast cancers and choice of surgical treatment: a retrospective study on a series of 1158 cases”

**DOI:** 10.1186/1471-2482-15-1

**Published:** 2015-01-14

**Authors:** Alessandro Neri, Daniele Marrelli, Tiziana Megha, Francesca Bettarini, Damiana Tacchini, Lorenzo De Franco, Franco Roviello

**Affiliations:** Unit of Surgical Oncology, Department of Oncology of the University Hospital of Siena, Siena, Italy; Unit of Pathology, Department of Oncology of the University Hospital of Siena, Siena, Italy; Department of Medicine, Surgery and Neurosciences of the University of Siena, Policlinico Le Scotte, v.le Bracci 14, 53100 Siena, Italy

**Keywords:** Breast cancer, Multifocal breast cancer, Multicentric breast cancer, Breast cancer surgery, Breast cancer prognosis

## Abstract

**Background:**

The biological and clinical significance of multifocal and multicentric (MF/MC) breast cancers and the choice of appropriate surgical treatment for these tumors are still debated.

**Methods:**

1158 women operated on for a stage I-III breast cancer were included in this retrospective study; clinical and pathological data were obtained from the institutional database of the Department of Oncology of the University of Siena, Italy. The impact of MF/MC breast cancers on patterns of recurrence and breast cancer specific survival (BCSS) was investigated in relation to the type of surgical treatment.

**Results:**

MF and MC cancers were present in 131 cases (11.3%) and 60 cases (5.2%) respectively and were more frequently treated with mastectomy (55 MF and 60 MC cancers, 81.2%) than with breast conserving surgery (36 MF cancers, 18.9%; p < 0.001). MF and MC breast cancers were associated with a worse prognosis with a BCSS of 154 months compared to 204 months of unicentric cancers (p < 0.001). In multivariate analysis, MF/MC cancers were independent prognostic factors for BCSS together with higher number of metastatic axillary nodes, absence of estrogen receptors and high proliferative activity. MF and MC cancers were related to a significantly shorter BCSS in patients submitted to mastectomy as well as those submitted to breast conserving surgery. Relapse at any site was higher in the subgroup of MF and MC cancers but the incidence of loco-regional and distant recurrences did not differ between patients treated with mastectomy or breast conserving surgery.

**Conclusions:**

Our results indicate that MF/MC cancers have a negative impact on prognosis and are related to higher loregional and distant relapse independently from the type of surgery performed. Adjuvant therapies did not modify the poorer outcome, but in patients receiving adjuvant anthacyclines, the differences with unicentric tumors were reduced. Our data support the hypothesis that MF/MC tumors may have a worse biological behavior and that the presence of multiple foci should be considered in planning adjuvant treatments.

## Background

The presence of multiple simultaneous foci of breast cancer has been a well-known issue for decades [1], and it was a deterrent for attempts to treat breast cancer with non-mutilating procedures until the publication of the first randomised trials of quadrantectomy and radiotherapy [2–4].

Breast cancers are defined as multifocal when there is more than one distinct tumour within the same quadrant of the breast (MF) and multicentric when multiple cancers develop in different quadrants of the breast (MC) [5]. MF/MC breast cancers have been reported with an incidence of 40–70% in serial-sectioning studies of mastectomy specimens [6–8], and are usually considered a contraindication to breast-conserving surgery [9] because of an increased risk of local recurrence [10, 11]. Indeed, breast-conserving surgery may be safely performed in the case of MF/MC cancer provided that the disease can be adequately excised with a good cosmetic result [12].

The real incidence of MF/MC breast cancer is unclear, as the presence of simultaneous cancers can be missed either at preoperative evaluation by mammography and ultrasound [13, 14] or at pathological examination, unless extensively searched with appropriate specimen analysis techniques [15].

Breast magnetic resonance imaging (MRI) has been shown to have a higher accuracy than conventional imaging in detecting MF/MC breast cancers [16]. Women undergoing preoperative MRI have a significantly higher proportion of MF/MC breast cancer diagnosed, and consequently a higher number of mastectomies performed [16, 17]. This aggressive surgical approach could improve the local control of disease and ultimately the survival, as recent studies show a possible worse prognosis for patients with locoregional relapse after breast-conserving surgery [18, 19].

However, such a tendency towards a more extensive surgery is debated. The COMICE trial did not show any reduction of local recurrence or any improvement of long-term survival in patients undergoing preoperative MRI [17]. Moreover, the presence of occult MF/MC breast cancer was a well-known issue when the first trials of conservative surgery were conducted. The similar survivals of patients treated with mastectomy or with breast conservation and radiotherapy in those trials may indicate that local control of the disease in the presence of MF/MC disease can be obtained with less extensive surgery without affecting long-term outcome [3]. As a consequence, the recent increase of mastectomies related to MRI diagnosis of MF/MC cancers is not justified [20].

The controversy remains when considering the post-surgical adjuvant treatments. In spite of the negative prognostic significance of MF/MC cancers suggested in early studies [8], the current edition of TNM stages breast tumours using only the diameter of the largest focus [21] and the presence of additional foci is not taken into account in deciding adjuvant therapies.

Indeed, the biological and clinical significance of MF/MC breast cancer is still controversial. In the literature, few studies have investigated the prognosis of MF/MC cancers, and they have produced contrasting results: some investigators have not found any influence on long-term survival [22–24] while other recent series have reported a worse outcome for MF/MC breast cancers [25, 26].

Therefore, it remains unclear whether MF/MC breast cancers should be considered a separate category with a potentially unfavourable impact on prognosis and whether these lesions require specific treatment with more extensive surgery or committed adjuvant therapies.

The present study was directed to analyse, in a large retrospective series of breast cancer patients treated at a single institution, the impact of MF/MC breast cancers on the long-term survival in relation to other known pathological and clinical factors and to the type of treatment received.

## Methods

From January 1991 to December 2005, 1478 women affected by breast cancer were operated on at the Department of Oncology, Section of Surgical Oncology, University of Siena, Italy. Clinical and pathological data of these patients were prospectively collected in an institutional computerised database; this database was approved by the Ethical Committee of the University Hospital of Siena and the data were registered upon informed consent of the patients. For the purposes of the present study, the Scientific Committee of the Department granted access to patient data contained in the database. We selected those patients without distant metastases at diagnosis that were submitted to primary surgery and axillary lymph nodal dissection. Patients with synchronous bilateral breast cancers were excluded from the study.

Follow-up data were collected from our outpatient clinic records, where the patients followed a standardised program of clinical and instrumental examinations; only patients with a follow-up of at least 36 months were included in the study.

As a result, the study population included 1158 cases of stage I–III breast cancers, with a median age of 63 years old (range 25–94) and 910 (78.6%) postmenopausal women.

A mastectomy was performed in 631 (55.5%) patients and a conservative surgery in 527 (44.5%); all patients submitted to partial mastectomy received radiation therapy on the residual breast with a boost on the tumour bed. In cases of preoperative diagnosis of MF/MC breast cancer, the primary surgical option was mastectomy, but a conservative surgical approach was proposed when adequate excision with acceptable cosmesis was possible. All patients included in the study received an axillary dissection of at least second level, with a mean number of 16 ± 6 nodes removed (range 9–34).

An adjuvant systemic treatment was administered to 923 (79.7%) patients, with 445 women receiving chemotherapy and 478 endocrine therapy. Hormonal therapy was adopted in 200 patients subsequent to chemotherapy.

Histopathological examination included: histological type of cancer, as defined according to the WHO Working Group classification [27]; pathological tumour stage, including tumour size and nodal status, assessed according to the criteria established by the TNM classification [21]; grade of tumour evaluated according to the Scarff–Bloom–Richardson classification modified by Elston and Ellis [28]; and presence of peritumoural lymphovascular invasion.

Estrogen receptors and progesterone receptors were assessed by immunohistochemistry and tumours were scored positive if at least 10% of tumour cells showed nuclear staining. Proliferative activity was evaluated by Ki-67 immunohistochemical assessment using a Mib-1 antibody; the cut-off value for high Mib-1 staining was chosen by semiquantitative analysis, and the value which maximised the separation of survival curves was 15% of neoplastic cells staining [29]. Her-2/neu was evaluated by immunohistochemistry giving a score range of 0–1+ (negative) to 3+ (positive); all 2+ scores were verified by fluorescent *in situ* hybridisation.

Breast cancers were defined as multifocal (MF) if there was more than one focus of invasive breast cancer separated by benign tissue in the same quadrant, and multicentric (MC) when distinct tumour foci were found in different quadrants of the breast. Surgical specimens were examined after slicing at 5-mm intervals. Each tumour focus was identified macroscopically. The surgical specimens were then fixed in 10% buffered formalin and processed into paraffin blocks for histopathological examination; normal tissue was confirmed to be present between the various tumour foci. Histology was reviewed by TM and DT.

Correlations between clinicopathological variables and MF and MC breast cancers were investigated by univariate analysis and the chi-square test was used to assess their statistical value; a p-value <0.05 was considered statistically significant. As the correlations between MF or MC cancers and clinicopathological variables were similar, these cases were grouped together and a logistic regression model was built in order to identify those factors independently associated with MF/MC cancers. Actuarial (Kaplan–Meier) breast-cancer-specific survival (BCSS) was calculated from the date of surgery. For the analysis of the events during follow-up, recurrence of disease was classified as local (breast or chest wall), regional (axillary, supraclavicular or internal mammary lymph nodes) and distant. We evaluated the prognostic significance of MF and MC breast cancers with respect to BCSS by means of a log-rank test. In order to compare such prognostic significance to that of the other clinical and pathological factors, a multivariate analysis was performed by means of Cox regression analysis; the model of regression included those factors that were significantly related to prognosis in univariate analysis. For statistical comparison, a p-value <0.05 was considered significant.

The Statistical Package for the Social Sciences software (version 16.0) (SPSS, Chicago, Illinois, USA) was used for statistical analysis.

## Results

We found a multifocal breast cancer in 131 cases (11.3%) and a multicentric breast cancer in 60 cases (5.2%). MF/MC breast cancers were more frequently treated with mastectomy (55 MF and 60 MC cancers, 81.2%) than with breast conserving surgery (36 MF cancers, 18.9%; p < 0.001). An adjuvant therapy was given in 79% (764 cases) of unicentric cancers and in 83.2% (159 cases) of MF/MC cancers (p = 0.138). Patients with MF/MC cancers more frequently received chemotherapy (103 cases, 64.8%) than hormone therapy (56 cases, 35.2%; p < 0.001), with a significantly higher administration of anthracycline-based regimens. The distribution of clinical and pathological factors in unifocal and MF/MC breast cancers is reported in Table 1.

**Table 1:** **Distribution of clinical and pathological factors in unicentric and multifocal/multicentric breast cancers**

		Multifocal/multicentric	Unicentric	p value
Age	≤40	14 (28.6)	35 (71.4)	p = 0.002
	41-50	45 (24.7)	137 (75.3)	
	51-60	33 (13.1)	218 (86.9)	
	61-70	43 (13.4)	279 (86.6)	
	71-80	43 (16.1)	224 (83.9)	
	>80	13 (14.9)	74 (85.1)	
Menopausal status	Premenopausal	58 (23.4)	190 (76.6)	p < 0.001
	Postmenopausal	133 (14.6)	777 (85.4)	
Histotype	Ductal infiltrating	123 (14)	755 (86)	p < 0.001
	Lobular infiltrating	44 (30.8)	99 (69.2)	
	Mucinous	9 (16.4)	46 (83.6)	
	Apocrine	5 (21.7)	18 (78.3)	
	Papillary	4 (19)	17 (81)	
	Tubular	3 (15.8)	16 (84.2)	
	Other	3 (15.8)	16 (84.2)	
Tumor size	1a (0.1 – 0.5 cm)	5 (9.4)	48 (90.6)	p < 0.001
	1b (0.6 – 1 cm)	18 (9)	182 (91)	
	1c (1.1 – 2 cm)	74 (15.3)	410 (84.7)	
	2 (2.1 – 5 cm)	56 (17.2)	269 (82.8)	
	3 (>5 cm)	38 (39.6)	58 (60.4)	
Nodal status	0	71 (10.2)	628 (89.8)	p < 0.001
	1a	49 (20.4)	191 (79.6)	
	2a	28 (21.2)	104 (78.8)	
	3a	44 (50.6)	43 (49.4)	
Tumor grade	1	40 (18.6)	175 (81.4)	p = 0.45
	2	100 (15.3)	553 (84.7)	
	3	51 (17.6)	239 (82.4)	
Lymphovascular invasion*	Absent	92 (12.3)	654 (87.7)	p < 0.001
	Present	88 (24.6)	269 (75.4)	
Estrogen receptors*	negative	76 (21.3)	280 (78.7)	p = 0.006
	positive.	105 (14.7)	611 (85.3)	
Progesteron receptors*	negative	63 (20)	252 (80)	p = 0.08
	positive	118 (15.6)	636 (84.4)	
Mib-1	≤15%	74 (14.6)	432 (85.4)	p = 0.4
	>15%	98 (17.5)	461 (82.5)	
Her-2/neu*	not overexpressed	99 (13.7)	625 (86.3)	p = 0.019
	overexpressed	46 (21.2)	171 (78.8)	

Number in parentheses are percentages; *missing cases: lymphovascular invasion 55, Estrogen receptors 86, Progesteron receptors 89, Mib-1 93, Her2/neu 217.

Multivariate analysis by means of logistic regression indicated that age lower than 50 years old, lobular histotype and higher number of metastatic axillary nodes were independently associated with MF/MC breast cancers (Table 2).

**Table 2:** **Factors independently related to multifocal/multicentric breast cancers in multivariate analysis (Logistic Regression)**

		RR	95% C.I.	p
Age class	<50 y.o.	1.66	1.099-2.51	0.016
Histotype	Infiltrating lobular	2.92	1.33-6.38	0.007
Nodal status	pN1	2.34	1.47-3.72	<0.001
	pN2	2.23	1.26-3.94	0.006
	pN3	8.67	4.84-15.52	<0.001

At a median follow-up of 88 months (range 11–248), we observed 304 disease recurrences with a median time to relapse of 32 months, and 172 patients died of breast cancer. Actuarial BCSS was 89.7% at 5 years and 79.8% at 10 years.

MF and MC breast cancers were associated with a worse prognosis with a BCSS of 154 months (95% C.I. 139–169 months) compared to 204 months (95% C.I. 194–214 months) of unicentric cancers (p < 0.001). Other clinical and pathological factors significantly associated with prognosis in univariate analysis are reported in Table 3.

**Table 3:** **Significant prognostic factors for breast cancer specific survival in univariate analysis**

		num.	5 yr BCSS* (%)	10 yr BCSS* (%)	p
Age	≤40	49	80.7	67.9	0.009
	>40	1109	90.1	80.3	
multifocal/multicentric breast cancer	No	967	91.7	82.7	<0.001
	Yes	191	79.4	72.5	
Tumor size	1a (0.1 – 0.5 cm)	53	100	83.3	<0.001
	1b (0.6 – 1 cm)	200	97.3	91.5	
	1c (1.1 – 2 cm)	484	92.3	83.9	
	2 (2.1 – 5 cm)	325	87.7	74.1	
	3 (>5 cm)	96	62.4	39.8	
pN	0	699	95.6	89.1	<0.001
	1 a	240	92.8	81.1	
	2 a	132	81	65.6	
	3 a	87	53	31.4	
Tumor Grade	G1	215	92	87	0.02
	G2	653	90.3	81.1	
	G3	290	86.5	67.5	
Lymphovascular invasion	Absent	746	93.4	84.1	0.001
	Present	357	81.6	69.7	
Estrogen receptors	positive	356	93.3	82	<0.001
	negative	716	82.3	74.2	
Mib-1	<15%	506	95.8	86.7	<0.001
	>15%	559	88.3	69.9	

*BCSS – breast cancer specific survival.

The prognostic impact of MF/MC breast cancers was confirmed when patients were stratified for tumour size and number of metastatic axillary lymph nodes (Table 4).

**Table 4:** **Prognostic value of multifocal and multicentric breast cancers according to tumor size and nodal status**

		5 year BCSS*		10 year BCSS*		
		Multifocal/multicentric	Unicentric	Multifocal/multicentric	Unicentric	
Tumor size	1a (0.1 - 05 cm)	100	100	80	100	p < 0.001
	1b (0.6 – 1 cm)	86.9	97.6	74.5	87.5	
	1c (1.1 - 2 cm)	84	93.7	73.3	84.6	
	2 (2.1 - 5 cm)	77.2	87.1	67.7	75.5	
	3 (>5 cm)	63.5	71.7	48.4	58.7	
Nodal status	pN0	90.7	96	74.9	89.4	p 0.02
	pN1	88.2	93.9	71.3	83	
	pN2	70.7	80.3	62.9	66.8	
	pN3	42	53.5	18.7	32.9	

*BCSS – breast cancer specific survival.

**Figure 1:**
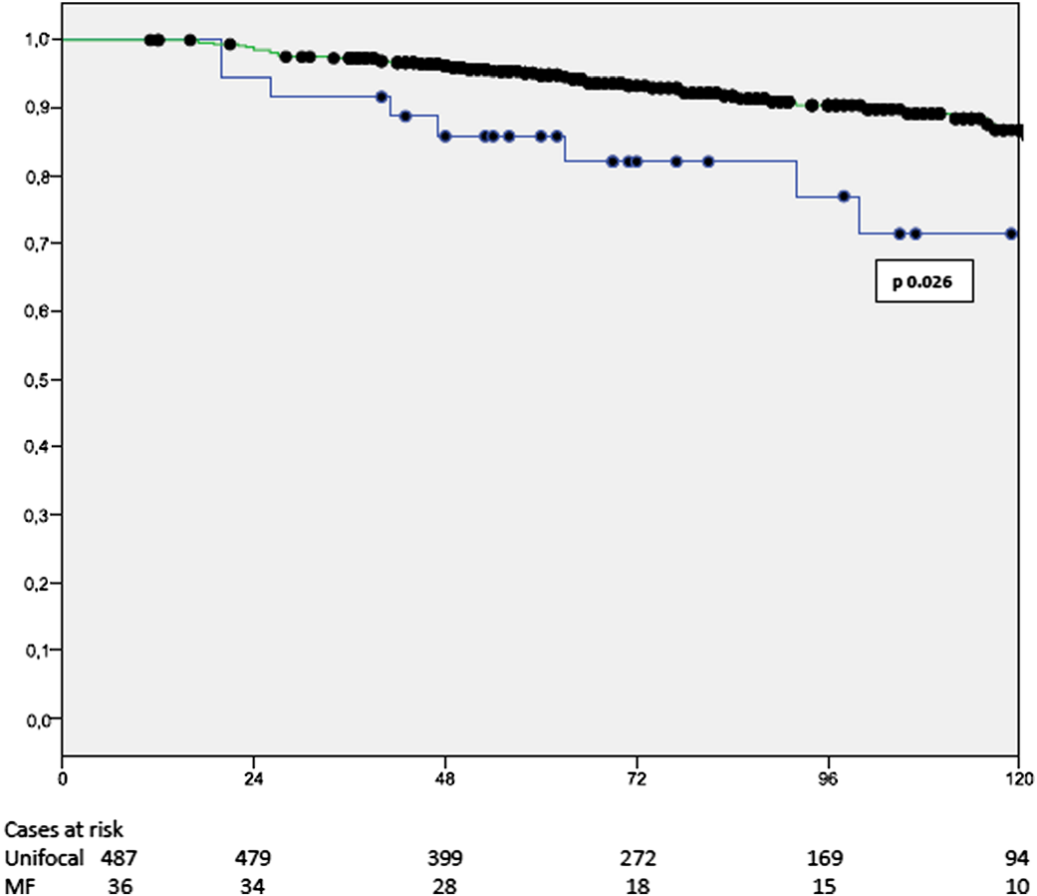
**Breast cancer specific survival in patients submitted to breast conserving surgery according to multifocality (Kaplan-Meier); x unifocal cancers, • multifocal (MF) cancers.**

**Figure 2:**
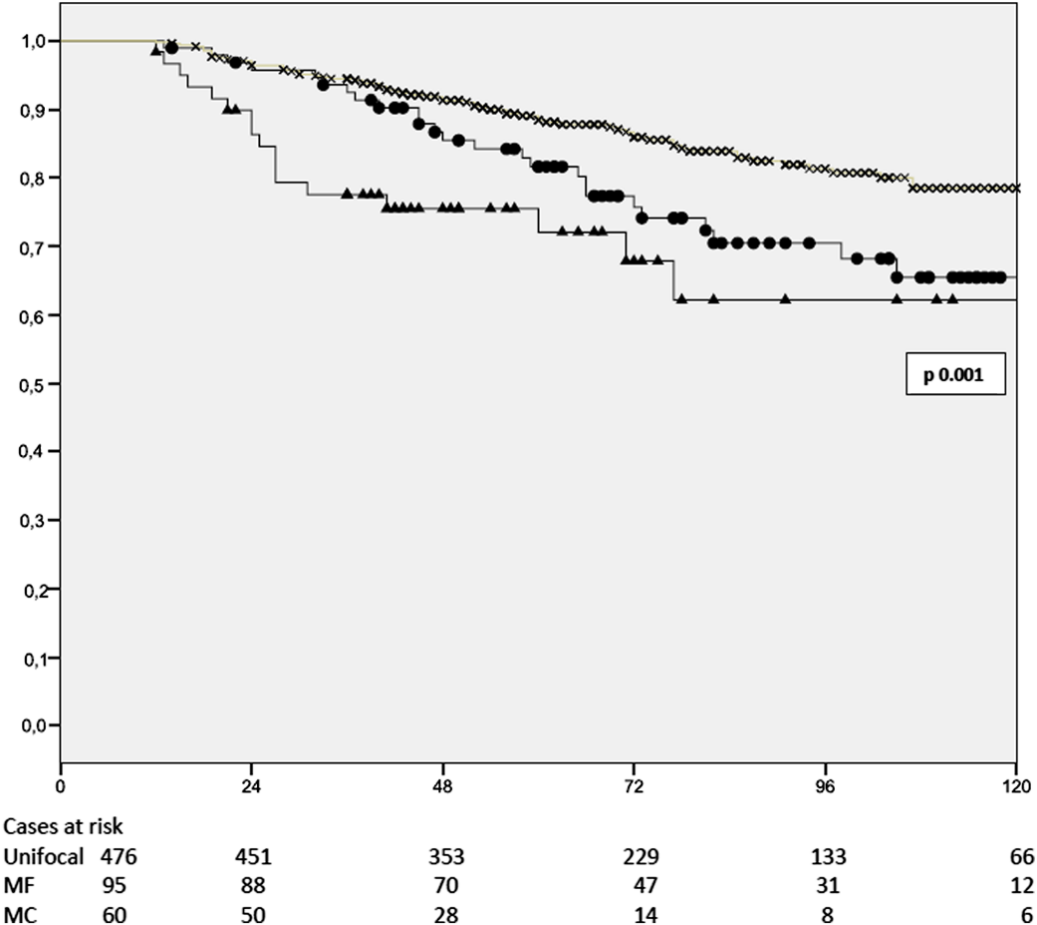
**Breast cancer specific survival in patients submitted to mastectomy according to multifocality and multicentricity (Kaplan-Meier); x unifocal cancers, • multifocal (MF), ∆ multicentric (MC) cancers.**

In multivariate analysis, MF/MC cancers were independent prognostic factors for poorer BCSS together with higher number of metastatic axillary lymphnodes, absence of oestrogen receptors and high proliferative activity as expressed by Mib-1 staining (Table 5).MF and MC cancers were related to a significantly shorter BCSS in patients submitted to mastectomy as well as those submitted to conservative surgery (Figures 1 and 2).

**Table 5:** **Multivariate Cox regression analysis for breast cancers specific survival in the whole population**

		HR	95% C.I.	p
multifocal/multicentric breast cancer	present	1.64	1.05-2.57	0.029
Estrogen receptors	absent	1.89	1.2-2.98	0.005
Mib-1	>15%	1.85	1.4-3.02	0.013
Nodal status	pN0	1		
	pN1	1.73	0.87-3.22	0.116
	pN2	3.08	1.53-6.16	0.002
	pN3	9	4.95-16.4	<0.001

In order to isolate the prognostic value of MF/MC breast cancers in mastectomy patients and breast-conserving surgery patients, a multivariate analysis was conducted in the two subgroups separately. Clinical and prognostic parameters significantly related with prognosis in univariate analysis were included in the regression model, and the prognostic impact of MF/MC cancers was adjusted for age, tumour size, nodal status, tumour grade, peritumoural lymphovascular invasion, oestrogen receptor status and Mib-1 expression. The results of this multivariate analysis are reported in Table 6 and confirmed the independent prognostic value of MF/MC breast cancers both in patients submitted to mastectomy and to breast-conserving surgery.

**Table 6:** **Multivariate Cox regression analysis for breast cancers specific survival in mastectomy and breast conserving surgery**

			HR	95% C.I.	p
Breast conserving surgery	multifocal/multicentric breast cancer	yes	3.88	1.06-14.12	0.02
	Mib-1	>15%	5.53	1.57-19.47	0.008
	Nodal status	pN1	3.01	0.98-9.28	0.055
		pN2	3.42	1.22-8.48	0.004
		pN3	16.8	5.31-36.4	<0.001
Mastectomy	multifocal/multicentric breast cancer	yes	2.72	1.15-6.48	0.023
	Estrogen receptors	absent	2.3	1.36-3.87	0.002
	Nodal status	pN1	1.56	0.68-3.59	0.28
		pN2	4.73	2.24-9.9	<0.001
		pN3	8.09	4.08-16.04	<0.001

The analysis of the sites of recurrence showed that relapse at any site was significantly more frequent in patients with MF/MC breast cancers. Table 7 reports the distribution of relapses in MF/MC breast cancers in relation to the type of surgery performed; the incidence of locoregional and distant recurrences in MF/MC cancers did not differ between cases treated with mastectomy or with conservative surgery.

**Table 7:** **Sites of breast cancer recurrence according to the type of surgery and the presence of multifocal and multicentric breast cancers**

		num.	Local		Regional		Distant	
Breast conserving surgery								
	unicentric	491	29 (5.9)	p 0.32	8 (1.6)	p 0.05	55 (11.2)	p 0.03
	multifocal/ multicentric	36	3 (8.3)*		2 (5.6)§		7 (19.4)°	
Mastectomy								
	unicentric	476	27 (5.7)	p 0.11	18 (3.8)	p 0.003	91 (19.1)	p 0.011
	multifocal/ multicentric	155	12 (7.7)*		11 (7.1)§		42 (27.1)°	
Total								
	unicentric	967	56 (5.8)	p 0.05	26 (2.7)	p 0.002	146 (15.1)	p < 0.001
	multifocal/ multicentric	191	15 (7.8)		13 (6.9)		49 (25.7)	

Number in parentheses are percentages.

*Difference statistically not significant between breast conserving surgery and mastectomy for local recurrence (p 0.92).

^§^Difference statistically not significant between breast conserving surgery and mastectomy for regional recurrence (p 0.63).

°Difference statistically not significant between breast conserving surgery and mastectomy for distant recurrence (p 0.31).

A significantly negative impact of MF/MC breast cancers on BCSS was present in patients who received an adjuvant treatment (either chemotherapy or hormone therapy), and in patients who did not receive any adjuvant treatment. A similar trend was also found in the subgroup of patients who received anthracycline-containing adjuvant therapy, with a 10-year BCSS of 61.7% and 53.7% for unicentric and MF/MC cancers respectively; however, this difference was not statistically significant (p = 0.065).

## Discussion

The reported incidence of MF/MC breast cancers in the literature ranges from less than 10% to more than 60% [8, 24]; such variability depends either on different definitions of multicentric and multifocal disease or on the techniques of breast specimen sampling used.

The 16.9% incidence of MF/MC breast cancers in our series is in line with data reported in other studies [22–24, 30, 31] that applied routine protocols of breast specimen sampling; a higher incidence of multiplicity is reported in whole-breast sectioning studies, but these techniques are usually reserved for research purposes [6–8]. However, in our experience, breast-conserving surgery was performed in almost half of the patients and this may have underestimated the real incidence of MF/MC as, in these cases, only the surgically removed quadrant was examined.

MF/MC cancers have been associated in the literature with younger age [24], large tumour size [24], lobular histotype [22, 25, 26], presence of peritumoural lymphovascular invasion [7, 25], and higher incidence of axillary nodal metastases [7, 8, 22, 24–26, 29]. Our study confirms the correlation between MF/MC breast cancers and clinical-pathological factors suggestive of more aggressive tumours, with the notable addition of the association with the absence of ER and Her2-neu positive status (Table 1). These relationships, particularly the higher incidence of nodal metastases, may indicate a different biological behaviour of MF/MC cancers with an increased propensity to disseminate, and consequently, a poorer prognosis [7, 30]. Indeed, the clinical significance of MF/MC breast cancers and their impact on prognosis are still debated. Vlastos et al. [22] studied a series of 284 women submitted to mastectomy and adjuvant therapy and did not find any prognostic value for MF/MC cancers, interpreting the presence of multiple tumour foci and related nodal metastases as markers of chronological age rather than biological aggressiveness of the primary tumour. The absence of prognostic significance of multiple foci of breast cancer on survival was confirmed by Litton et al. [23] in 300 young women aged less than 35 years old, and in the review on 7024 cases from the Danish Breast Cancer Group [24], where the significant association with a reduced disease free survival was explained by the increase in local recurrence, which did not affect BCSS, in the subgroup of patients with multifocal disease submitted to conservative surgery.

Our study is based on the records of patients prospectively collected in a dedicated database, and the design may then be considered similar to an observational study. Moreover, follow-up has been conducted at our outpatient clinic with an accurate prospective collection of information on the sites of relapse, which are usually not reported in other similar studies in the literature.

The majority of MF/MC cancers in our series were treated by mastectomy. This is an issue common to similar studies, because the presence of multiple foci often excludes the possibility of breast-conserving surgery and because pathological examination of the whole breast allows discovery of a higher number of MF/MC cancers. Even if we analysed the prognostic influence of MF/MC cancers treated with mastectomy and conservative surgery separately, this aspect may be a limitation of our study.

Our results indicate that MF/MC breast cancers have a negative independent impact on BCSS, and this finding is in line with other recent papers. In the largest cohort of 25,320 patients reported in the literature, Yerushalmi et al. [25] found an independent negative prognostic value of MF/MC breast cancers on outcome, even if of low impact. The same results were recently reported by Weissenbacher et al. [26] in a matched-pair analysis that included 288 pairs of unifocal vs. MF/MC breast cancers, which demonstrated that multifocality and multicentricity were independent predictors of reduced BCSS and increased local relapse and distant metastases.

We investigated the prognostic impact of MF/MC breast cancers in relation to the different types of breast surgery performed. The negative prognostic impact of MF/MC cancers on BCSS was present in both cases treated with conservative surgery and with radical surgery. MF cancers treated with conservative surgery had an ipsilateral breast recurrence rate of 8.3%, higher than the rate (5.9%) of unicentric cancers, but similar to the 7.7% incidence of local recurrences of MF/MC cancers submitted to mastectomy. MF/MC cancers were related to increased locoregional and distant relapses independently from the type of surgery performed, and the median BCSS for MF/MC did not differ between patients treated with breast-conserving surgery (177.4 months, 95% C.I. 150.3–204.5) and with mastectomy (145.19 months, 95% C.I. 128.6–161.7; p = 0.148). These findings are in accordance with the hypothesis that a more aggressive surgical approach does not improve either the locoregional control or the distant outcome of MF/MC tumours [20].

When analysing BCSS with respect to adjuvant chemotherapy, we found that neither hormone therapy nor chemotherapy modified the poorer outcome of MF/MC cancers; nonetheless, in patients receiving adjuvant anthracyclines, the differences in outcomes were reduced and lost statistical significance. These results are similar to those reported by Weissenbacher [26], who described a statistically significant worse outcome in MF/MC cancers treated with hormone therapy and a similar but not significant trend for cases treated with chemotherapy.

Indeed, dividing our series on a chronological basis (5-year periods of study), we found that the 10-year BCSS varied from 83.8% for unifocal and 60.6% for MF/MC breast cancers (p < 0.001) in the first period of study, to 91.5%, for unifocal and 84.5% for MF/MC (p = 0.035) in the last period. This reduced difference over time may be related to the increased number of patients with MF/MC cancers submitted to adjuvant chemotherapy with anthracyclines (from 16.9% in the first period to 42.2% in the last period). This is in accordance with the study of Vlastos [22], which suggests that adjuvant chemotherapy, particularly the more intensive regimens including anthracyclines, may be effective in reducing the negative impact of multiplicity.

The current TNM classification considers only the dimension of the largest tumour focus, with a possible underestimation of the higher tumour burden of MF/MC cancers. Some authors have proposed that the aggregate diameter of MF/MC tumours may be an effective estimate of the tumour load [30] and should be considered when planning the treatment of these patients, but these data were not confirmed in other studies [31]. More detailed analyses of tumour size using aggregate volumes of surface areas showed that MF/MC breast cancers have a higher incidence of nodal metastases than unicentric cancers of similar volume [32]. These results support the hypothesis that MF/MC breast cancers have a worse biological behaviour than unicentric tumours, independently from tumour burden.

## Conclusions

Despite the limitations of a retrospective study, our experience supports the hypothesis that MF/MC cancers are biologically more aggressive than unifocal tumours, have an increased propensity to metastatic diffusion and are related to a worse outcome.

The analysis of the patterns of recurrence suggests that breast conservation can be safely performed in MF cancers, provided that an adequate excision is warranted, and that more aggressive surgical approaches do not result in a better locoregional control or in reduction of distant recurrences.

The increased aggressiveness of MF/MC breast cancers may be faced with committed systemic adjuvant therapies; as a consequence, we suggest that the presence of MF/MC cancer should be considered in planning the adjuvant treatments to convey the increased risk.

## Abbreviations

MF/MC: Multifocal and multicentric
BCSS: Breast cancer specific survival.
